# Supplementary material for: PMEPA1/TMEPAI Is a Unique Tumorigenic Activator of AKT Promoting Proteasomal Degradation of PHLPP1 in Triple-Negative Breast Cancer Cells
Source: Cancers (Basel). 2021 Sep 30;13(19):4934. doi: 10.3390/cancers13194934 (PMC8508116; doi:10.3390/cancers13194934)
Supplement: Supplementary file 1 [file cancers-13-04934-s001.zip › cancers-1365731-supplementary.pdf]

Supplementary Materials: PMEPA1/TMEPAI is a Unique Tumorigenic Activator of AKT Promoting Proteasomal Degradation of PHLPP1 in Triple-Negative Breast Cancer Cells

Md. Anwarul Haque, Mohammed Abdelaziz, Meidi Utami Puteri, Thanh Thao Vo Nguyen, Kosei Kudo, Yukihide Watanabe and Mitsuyasu Kato

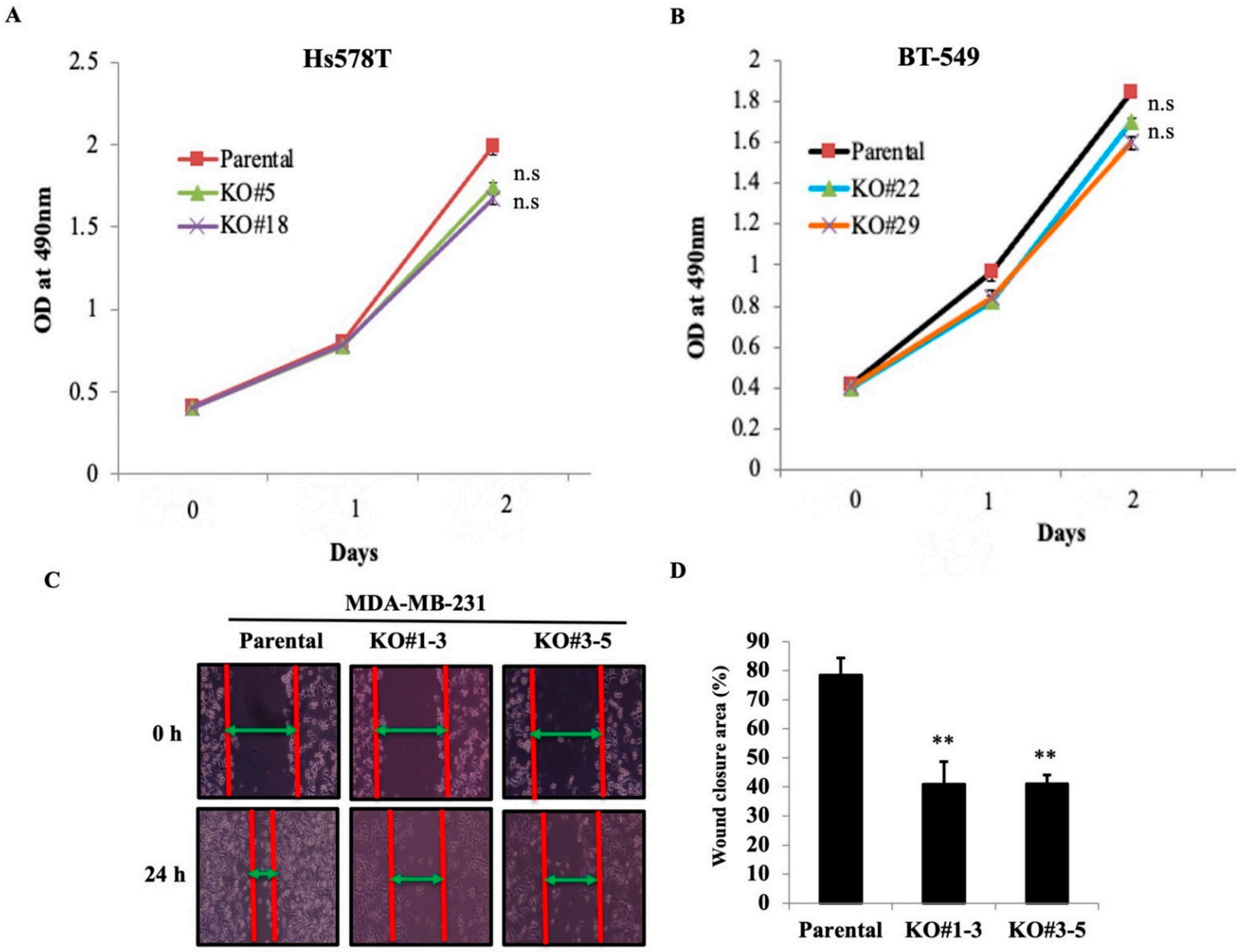

Figure S1. Role of TMEPAI on controlling cell proliferation and migration. (**A**) and (**B**), Hs578T and BT-549 cells (both parental and TMEPAI KO), respectively were seeded in 96-well plate and cultured for 24 and 48 hours, then MTS reagent was added to each of the well and incubated for 2h. Following incubation, absorbance of 490 nm was measured. The "n.s." means not significant. (**C**), MDA-MB-231 parental and TMEPAI KO cells were cultured at 80% confluency then scratched would was created. The photos were taken at 0 and 24 hours after scratching. (**D**), Bar chart depicts statistical three biological experiments. The asterisk symbols indicate significant difference from the parental at $p \leq 0.01$ (**).

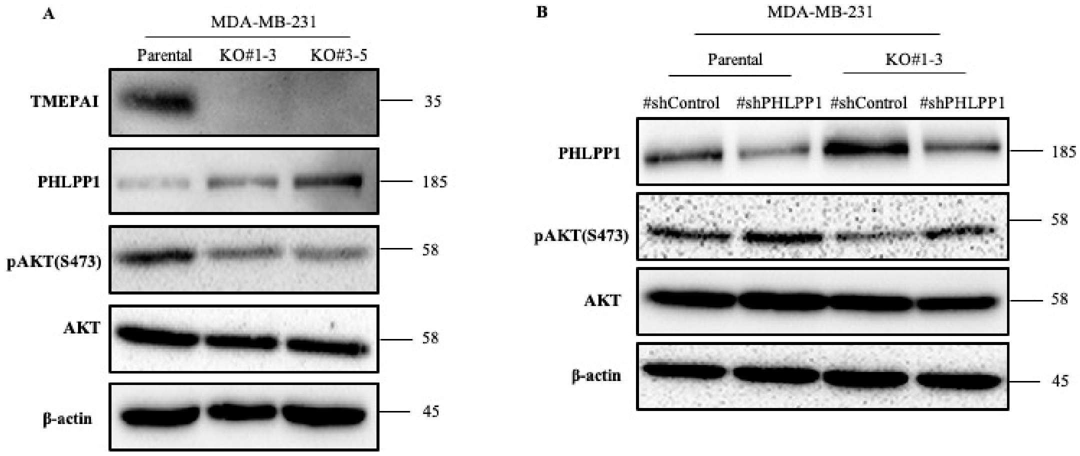

Figure S2. TMEPAI regulates AKT phosphorylation by suppressing PHLPP1. (**A**), Lysates from TGF-β stimulated parental and TMEPAI-KO MDA-MB-231 cells were subjected to Western blot analysis to detect TMEPAI, PHLPP1, pAKT(S473), and AKT. (**B**), Stable PHLLP1 KD efficiency was confirmed, and the amounts of pAKT(S473) and AKT in MDA-MB-231 cells were detected by use of Western blot analysis. β-actin was used as the loading control.

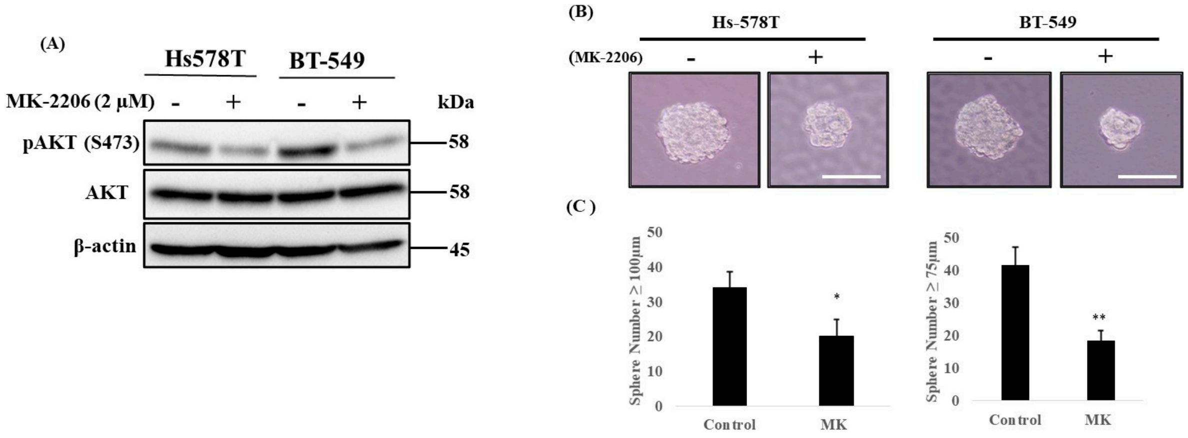

Figure S3. AKT allosteric inhibitor (MK-2206) suppresses AKT Ser473 phosphorylation and inhibits tumor-sphere formation. (**A**), Lysates from MK-2206 treated and untreated TNBC cells (Hs578T and BT-549) were prepared and subjected to western blot analysis to detect pAKT(S473), and total AKT. (**B**) and (**C**), Tumor sphere-forming ability of TNBC cells (Hs578T and BT-549) in the presence or absence of AKT inhibitor. The scale bar indicates 100 µm. The bar charts depict the numbers of large spheres formed by the parental and TMEPAI-KO cells (Hs578T and BT-549, respectively). The values presented here are the means ±SDs of 3 independent experiments. The bars with asterisks indicate significant differences between the parental cells at $p \leq 0.05$ (*) or $p \leq 0.01$ (**).

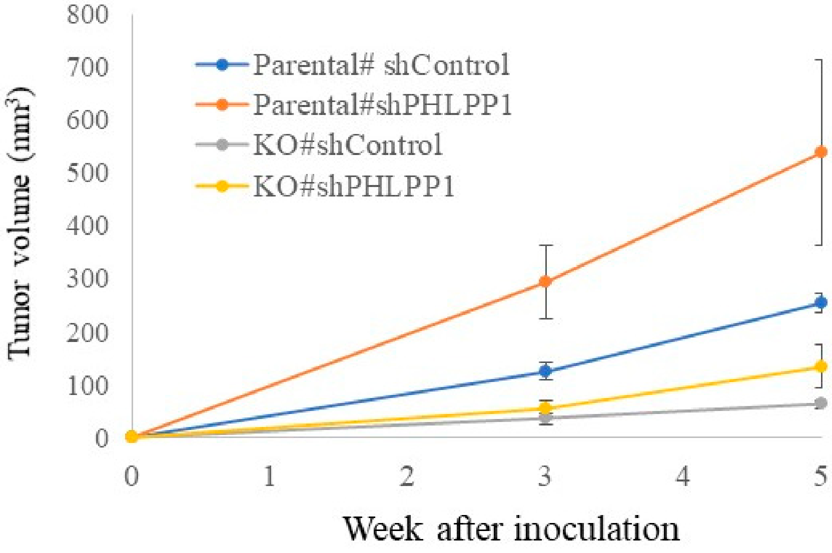

Figure S4. PHLPP1 KD increased in vivo tumorigenicity in both parental and TMEPAI KO cells. Stable PHLPP1 KD and control cells from both parental and TMEPAI KO MDA-MB-231 cells were subcutaneously injected to Balb/c nude mice and grossly observed tumors were measured with caliper at 3 and 5 weeks after inoculation. Tumor volume was calculated from the formula [(long diameter × short diameter × short diameter × π)/6].

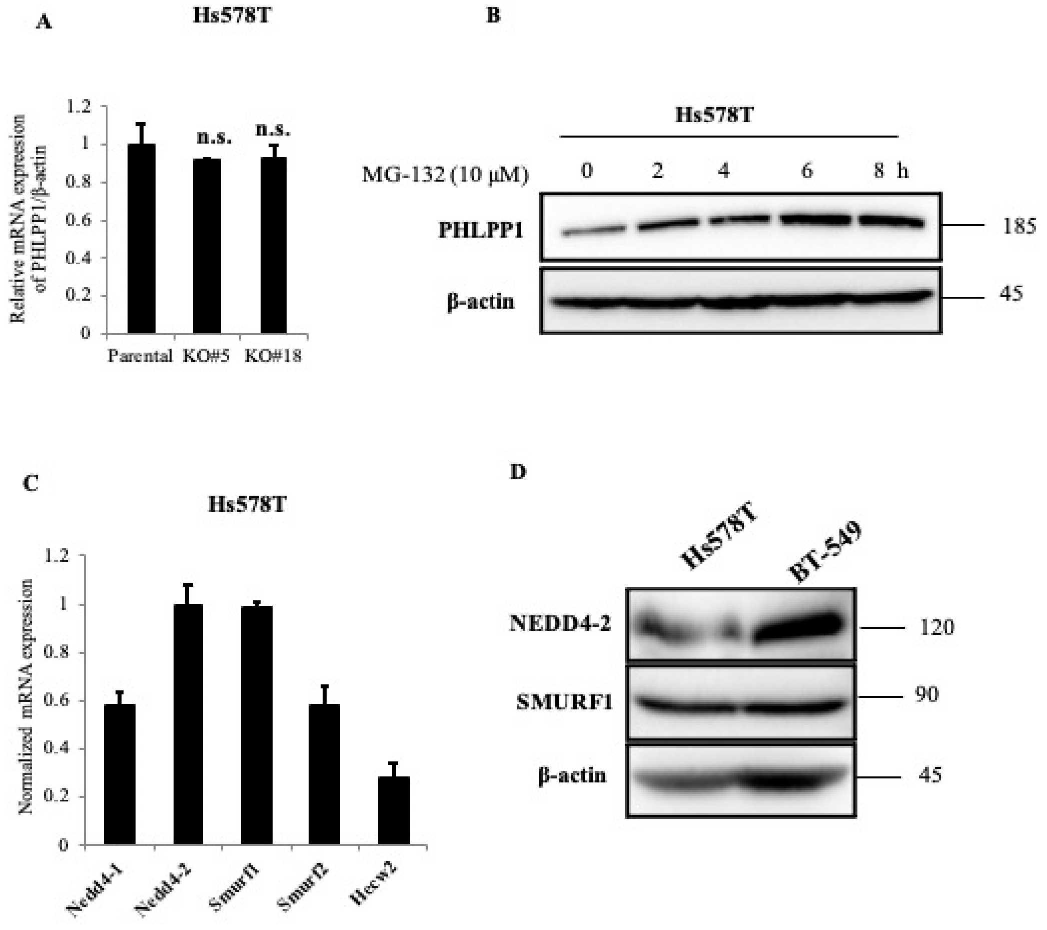

Figure S5. PHLPP1 degradation pathway and different HECT E3 ligase expression in TNBC cells. (**A**), PHLPP1 mRNA expression levels in both parental and TMEPAI KO Hs578T cells were measured by use of RT-qPCR. (**B**), Accumulation of PHLPP1 protein by a proteasomal inhibitor was detected at different time intervals. (**C**), The mRNA expression levels of different HECT type E3 ligases in Hs578T were quantified by use of RT-qPCR. (**D**), The protein levels of NEDD4-2 and SMURF1 were further confirmed in two different TNBC cell lines by use of Western blot analysis. β-actin was used as the internal loading control.

Raw WB data for Fig. 3 (A)

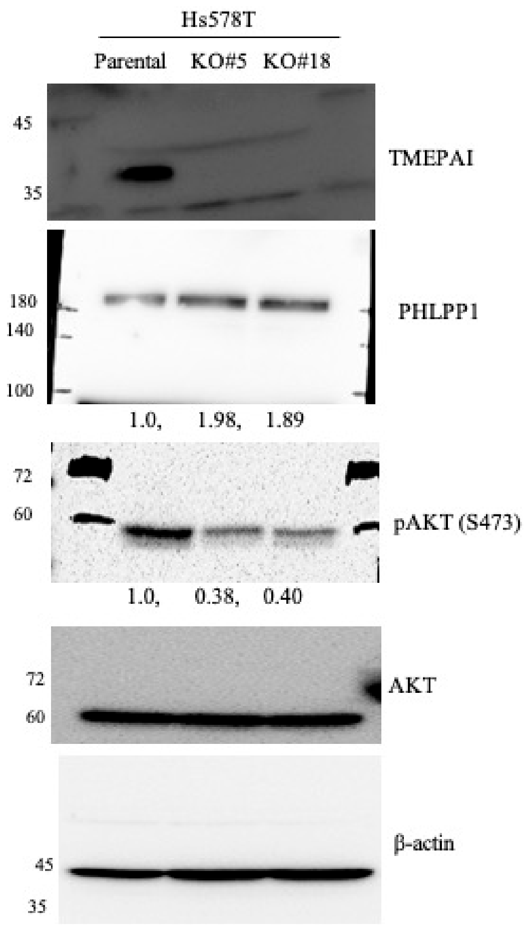

Raw WB data for Fig. 3 (B)

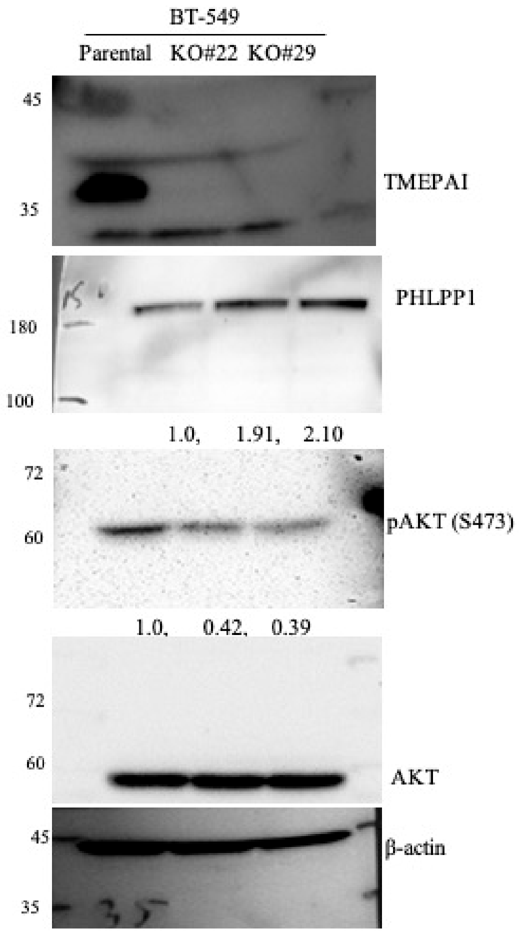

Raw WB data for Fig. 3 (C)

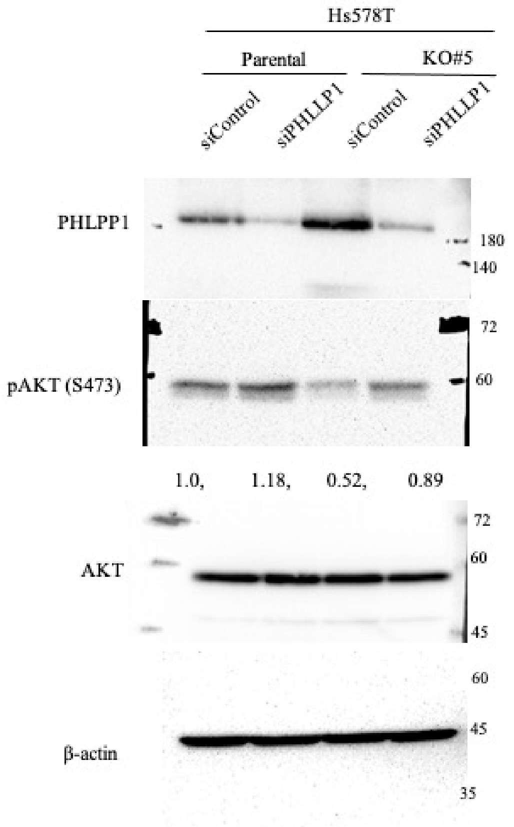

Raw WB data for Fig. 3 (D)

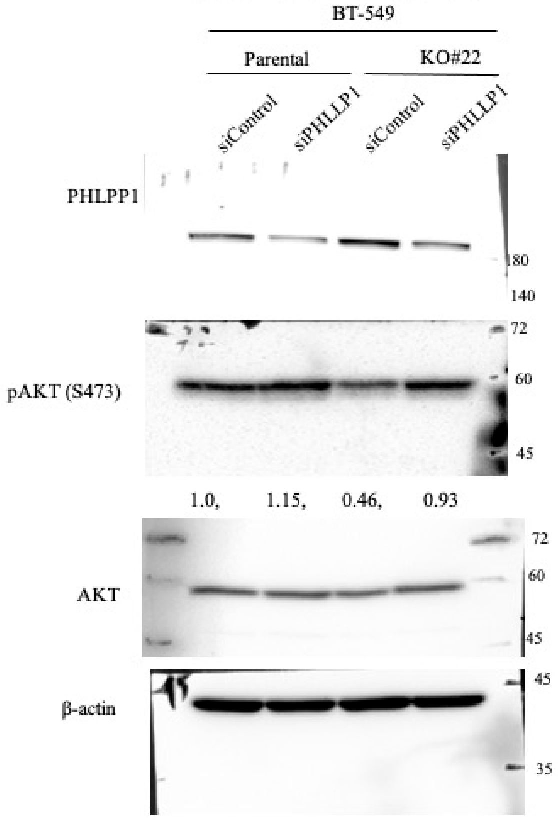

Raw WB data for Fig. 5 (C)

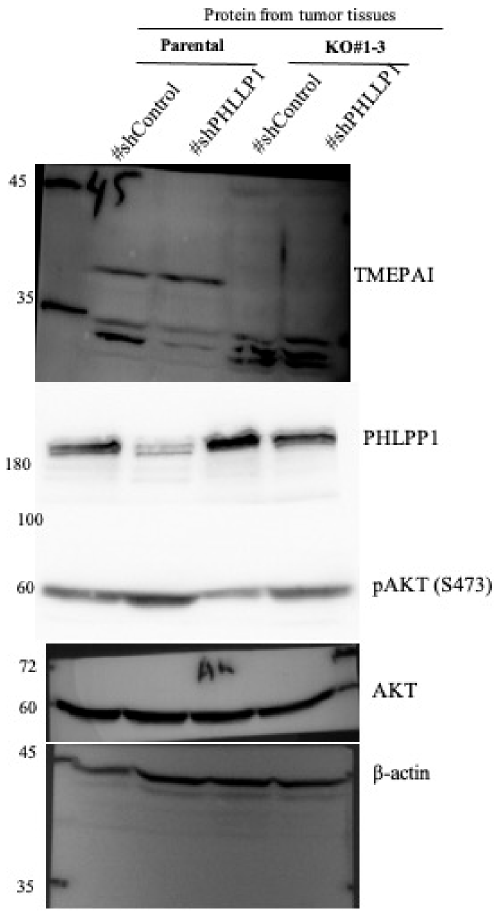

Raw WB data for Fig. 6 (B)

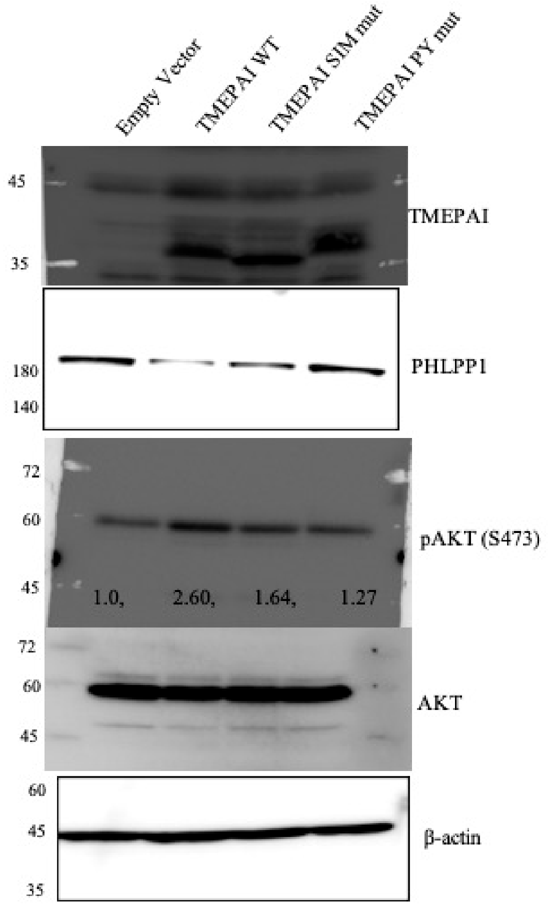

Raw WB data for Fig. 7(A)

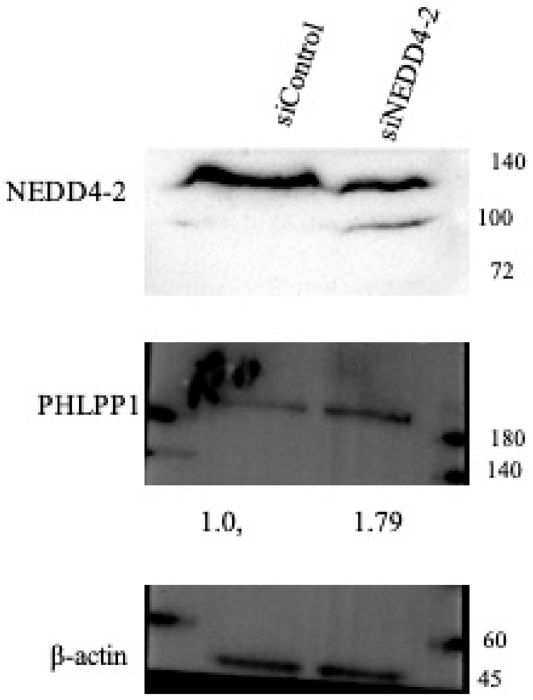

Raw WB data for Fig. 7(B)

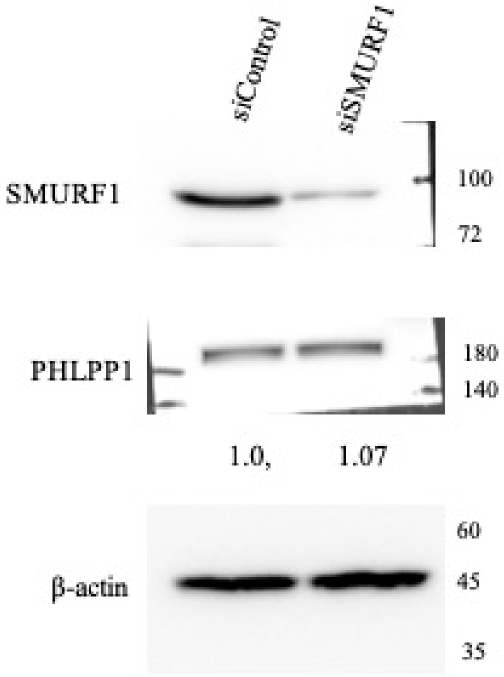

Raw WB data for Fig. 7(C)

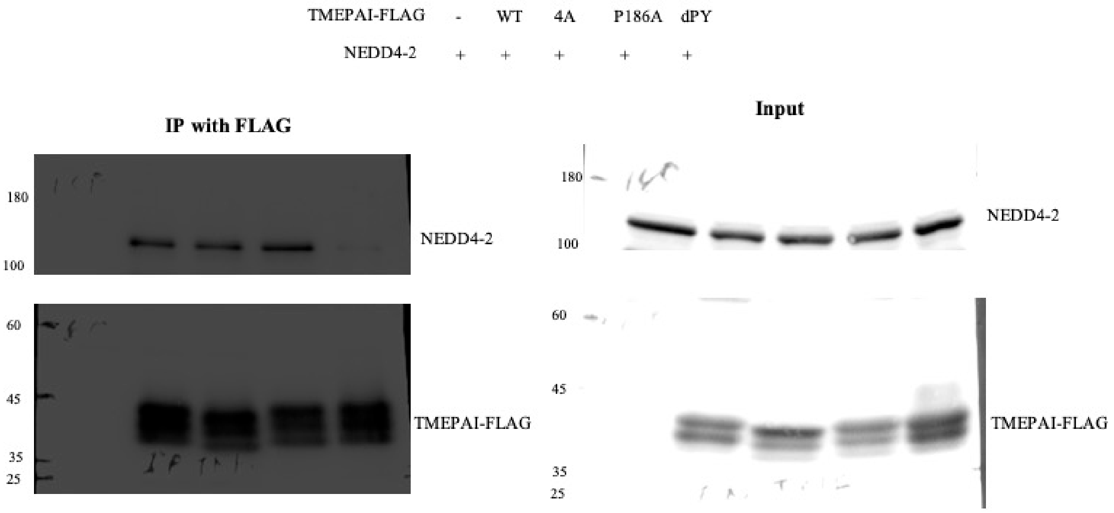

Raw WB data for Fig. 7 (D)

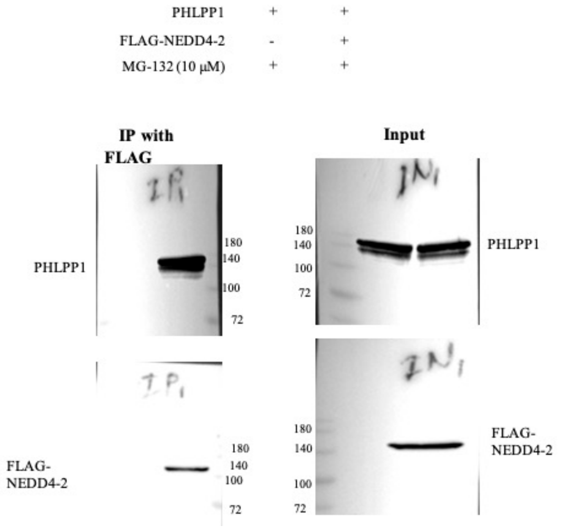

Raw WB data for Fig. 7 (E)

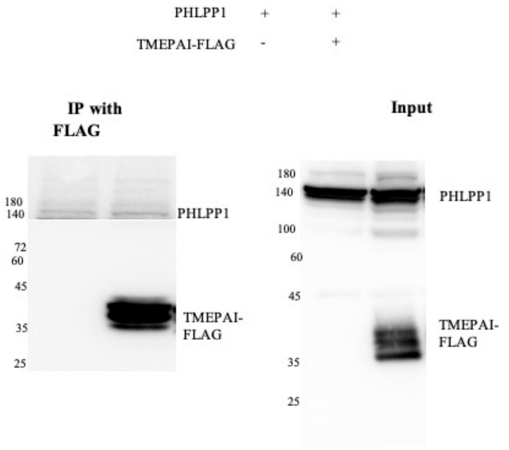

Raw WB data for Fig. 7 (F)

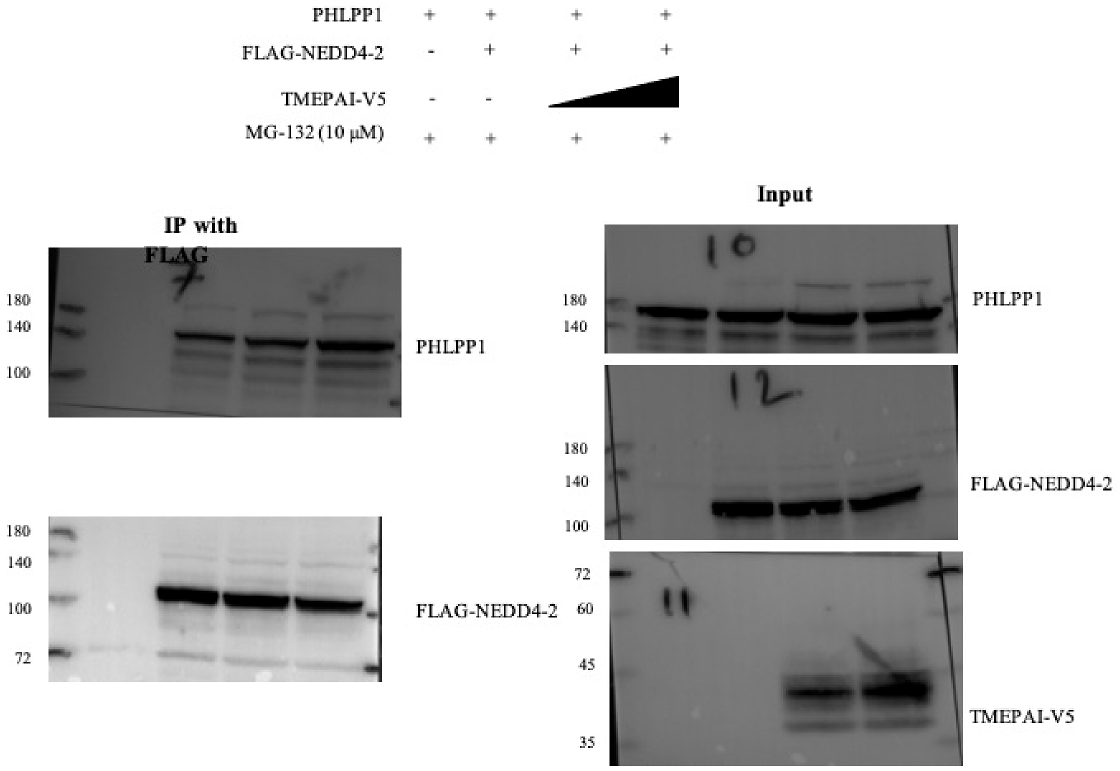

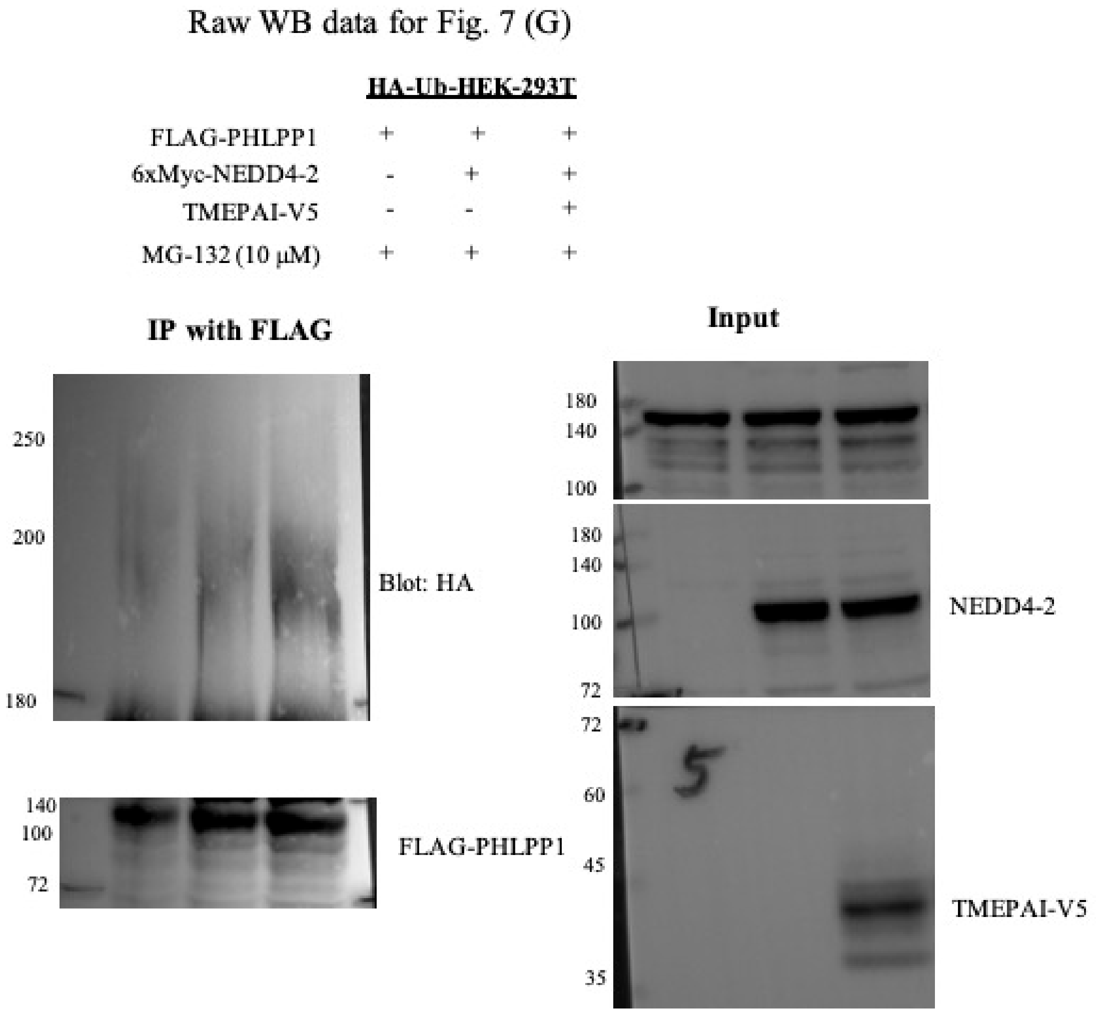

Figure S6. Uncropped Western Blot images.
